# The Effect of Packaging Color and Health Claims on Product Attitude and Buying Intention

**DOI:** 10.3390/ijerph17061991

**Published:** 2020-03-18

**Authors:** Alexandra Theben, Melissa Gerards, Frans Folkvord

**Affiliations:** 1 Doctoral Programme Information and Knowledge Society, Universitat Oberta de Catalunya, 08035 Barcelona, Spain; 2 Behavioral Science Institute, Radboud University, 6500 HE Nijmegen, The Netherlands; mpa.gerards@gmail.com; 3 Tilburg School of Humanities and Digital Sciences, 5037 AB Tilburg, The Netherlands; fransfolkvord@gmail.com

**Keywords:** food cues, food marketing, food packaging, healthy food

## Abstract

Packaging design is an important factor when consumers look out for healthy food. The study tested for effects of packaging color and health claims of a fictional fruit yoghurt package on attitude towards the product and subsequently, consumer’s buying intention, using a 2 × 2 between-subjects experimental design. We also tested whether interest in healthy food is a moderating factor. We found no evidence to support that visual cues (color) and textual cues (health-related advertising claims) are effective in influencing consumer attitude towards the product. Consumers did not show a more positive attitude towards products presented in low-arousal packaging colors (green/blue) compared to high arousal packaging colors (red/yellow). Also, the claim “palatability” did not result in a more positive attitude towards the product than the claim “healthy”. A moderating role of interest in healthy food could not be confirmed. The results confirmed, however, a significant relation of attitude towards the product and buying intention. Thus, buying intention could be explained mostly by whether consumers had a positive or negative attitude towards the product, which confirms that people’s attitudes are powerful predictors of buying decisions.

## 1. Introduction

The increased intake of processed and calorie-dense foods has led to increasing rates of overweight and obesity across countries worldwide, including the European Union [1]. While multiple factors are related to obesity, the fundamental cause of obesity is the energy imbalance between calories consumed and calories expended [1,2,3]. As suggested by the World Health Organization [1] obesity and related morbidities can be prevented through supportive environments that shape people’s choices, for example by making the selection of healthier food items to be consumed the easiest choice for consumption. 

An increasing number of consumers is looking for products that reflect health and well-being; usually products low in fat, sugar, salt, or calories [4,5]. Consumers often seek out claims on product packaging that fall in line with their general goals of wellness and wellbeing. This culminated in the rise of so-called “better-for-you” (BFY) products, which are often advertised as either low in fat or completely fat-free, typically contain artificial sweeteners instead of sugar, or have added fibers or vitamins [6]. A number of studies suggest, however, that despite the fact that BFY products often fare better than other products in terms of nutritional quality, they often contain high levels of sugar [7,8]. Ever since their market introduction, BFY products have gained much popularity given their strong association with a healthy lifestyle and the societal interest in eating healthier [4,9]. A large proportion of sugar-containing products use labels that position them as healthy despite containing high levels of sugar [8].

Changing the packaging design, adding information on labels, or even altering brand names are commonly used strategies to guide health-conscious consumers towards these BFY products [10,11]. In particular, food packaging helps consumers to intuitively assign food into categories such as “healthy” and “unhealthy” [12,13,14]. In relation to product packaging, it means that health-related claims like “*low in fat*”, “*lowers cholesterol*”, “*without added sugars*” or “*good for your bones*”, or even certain images or packaging colors that denote health, induce consumers to believe that the product would be good for them and as a consequence, influence purchasing behavior [10,15]. Yet, it remains unclear how, and on the basis of what type of information, consumers perceive products to be more or less healthy. It further remains unclear if these different perceptions affect their attitudes toward the product and subsequently, the intention to consume the product. 

A theoretical model that aims to explain the effects of food cues on consumption behavior is the Reactivity to Embedded Food Cues in Advertising Model (REFCAM) [1]. Studies have shown that exposure to cues of palatable foods can lead to consumption by activating automatic eating responses—both physiological and psychological [1,16,17,18]. This reactivity to food cues is difficult to suppress, in particular among individuals that are susceptible to these food cues [1,19,20,21,22]. More specifically, health-related cues on food packaging can lead to the formation of positive attitudes towards the brand and product, for example, related to its perceived healthiness [23,24]. 

Also, the theory of planned behavior provides support to our understanding of health-related behaviors [25]. As the theory of planned behavior suggests [25], intentions to perform behaviors of different kinds can be predicted with high accuracy from attitudes toward the behavior. More concretely, the primary determinant of health-related behavior is a person’s intention to carry out that particular behavior. It is therefore important to assess the impact of packaging on attitudes towards the product and subsequently the intention to consume.

Previous research found effects of food cue exposure on high-caloric food intake, but studies about the direct relationship between consumers’ responses to packaging cues that convey symbolic meaning, specifically healthiness, and buying intention have received little attention. The concept of packaging design includes many different elements such as shape, logo design, size, colors, illustrations, material, and nutrition information [26]. This study focusses on two kinds of packaging cues that differ in their ability to attract attention and communicate information, but which are both able to convey symbolic meaning about the product’s healthfulness, namely visual cues (with a focus on color) and textual cues (with a focus on advertising claims) [14,27,28,29]. Most existing studies studying the impact of cues on food packaging on consumers’ buying intention focused on unhealthy food products [1,12,29,30]. 

In addition, individual susceptibility factors have not been established extensively, such as food knowledge [31] and consumers’ interest in healthy food [30] although they should be examined in more depth in order to improve our understanding of the exact mechanism of how food advertising affects eating behavior [1]. The aim of the present study is to fill this gap and examine the effects of health cues on food packaging on buying intention, and to assess whether individual susceptibility factors may serve as possible moderators. Ultimately, we seek to extend knowledge on how consumers can be motivated to consume more healthy food and hence, improve health outcomes. 

Palatability is regarded as a hedonic attribute (i.e., feeling of pleasure), and is often associated with unhealthy foods, which stimulate more intake than others. Healthfulness attributes in turn are associated with a healthy lifestyle and wellbeing [32]. The current study makes use of different color and health-related claims as design variables, because they are often modified to impact consumer perceptions [13,29]. As most previously conducted studies only tested the effects of packaging in relation to unhealthy foods, this study provides interesting insights in relation to BFY food products. Previous research investigating the effects of packaging design confirmed the relation between health impressions and color variations [29]. More specifically, it showed that high-arousal colors (red/yellow) are associated with ’unhealthy’ foods, while low-arousal colors (green/blue) are associated with “healthiness” [29].

Based on these findings, the current study made use of green/blue colored packaging design to make the package appear healthy (health claim). Meanwhile, red/yellow colored packaging were designed to appear tasty (palatability claim). Previous research on how nutrition/health claims affect consumer perceptions and purchase decisions [33,34] were used to design food packaging with a “healthy” versus a “hedonic” (tasty) advertising claim. Hence, the claim “healthy” was used as a healthiness attribute, while “tasty” was used as a hedonic attribute.

The present study contributes to existing literature about the influence of product packaging design and existing health concerns by examining the influence that cues on food-packaging have on attitude towards the product and subsequently, consumer’s buying intention. We expected that participants who saw a product with a low-arousal packaging color would have a more positive attitude towards the product compared to participants who saw a product with high-arousal packaging colors (H1a). Furthermore, we expected that participants who saw a product with a palatability claim would have a more positive attitude towards the product compared to participants who saw a product without a palatability claim (H1b). We expected these effects to be moderated by interest in healthy food (H2). Lastly, participants with a more positive attitude towards the product were expected to have a stronger buying intention than participants with a less positive attitude towards the product (H3).

## 2. Materials and Methods 

### 2.1. Experimental Design and Stimulus Material 

**Figure 1:**
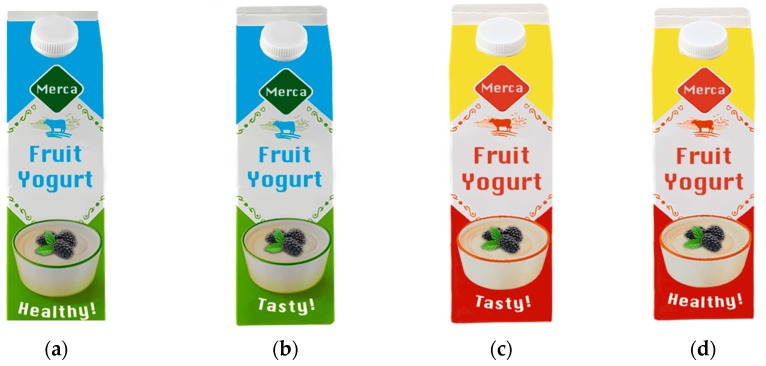
Visualization of the four conditions. (**a**) Healthy colors, health claim. (**b**) Healthy colors, palatability claim. (**c**) Unhealthy colors, palatability claim. (**d**) Unhealthy colors, health claim.

The present study used a 2 (packaging color: low-arousal colors vs. high-arousal colors) × 2 (advertising claim: Health claim vs. palatability claim) between-subjects experimental design using an online survey to examine the effect that health claims on food packaging have on consumers’ buying intention. The study was approved by the Behavioural Science Institute committee of the Radboud University. Participants (*N* = 300) were randomly assigned to one of these four conditions by stratified random sampling to avoid within-groups variation that causes systematic differences [33]. For the manipulation of this experiment, four visuals of a fictional fruit yoghurt package were created using Adobe Photoshop CC 2017, version 18.0.1 (see Figure 1). The dimensions of the products remained equal for all products. 

We created fictional brands in order to avoid prior knowledge of the nutrient levels in consumer’s typically consumed products. This eliminated the probability that participants would make a choice based on the reputation of or preference for a specific brand instead of the manipulation. Fruit yoghurt as a “better-for-you product” (BFY) was chosen, because fruits and yogurt are considered as functional foods that contribute to a healthier lifestyle [13]. Especially skimmed and semi-skimmed yoghurt are often typified as “healthy” foods [35]. A combination of fruit and yogurt should therefore be perceived as even healthier. However, fruit yoghurt contains far more sugar than is generally assumed. Usually, yoghurts contain between 8.7 and 12.9 g of sugar per 100 g [35]. This means that fruit yogurt contains high amounts of refined sugar and other additives, despite being advertised as healthy [6].

### 2.2. Participants

The participants were recruited using convenience sampling. Participants were recruited through a shared link of the questionnaire through social media. The age range was between 18 and 71 years old, with a mean of 36 years and standard deviation of 13.01 years. Half of the participants was male, and 55% was higher educated, while 45% was lower educated. We conducted a power analyses (G*Power [36]), that suggested that *N* = 199 was expected to be sufficient to detect significant (α = 0.05), medium effects (f = 0.20) of conditions (power = 0.80). Nonetheless, we aimed for 300 participants in order to conduct sensitivity analyses.

### 2.3. Procedures 

All participants received a short introduction to the research topic explaining the study on the welcome screen of the online questionnaire. All participants agreed to participate voluntarily, and at any time and for any reason, could refuse to answer a question or stop filling out the questionnaire.

After agreeing to participate in the experiment, participants were randomly allocated to one of the four conditions. The first screen of the questionnaire instructed them to imagine themselves shopping in the dairy isle of a local a supermarket where they encountered the yoghurt presented. Participants subsequently submitted information on their attitude towards the product. As a next step, they entered personal information, including interest in healthy food, gender, education level, and age. Finally, participants were thanked again for their cooperation. 

### 2.4. Measures

All measures in the questionnaire were rated on 7-point Likert scales, ranging from 1 (strongly disagree) to 7 (strongly agree) [14]. A 7-point scale has been chosen, as it enables participants to gradually distinguish the intensity of their feelings. Also, it is the most used technique within marketing research [37].

#### 2.4.1. Attitude Towards the Product 

We adapted a scale from MacKenzie and Lutz [38] and Singh and Cole [39] to measure attitude towards the product. The ten bi-polar items were based on the question “What did you think of the product you just saw?”. The respondents could answer on a 7-point-scale with “dislike very much/like very much”; “useless/useful”; “worthless/valuable”; “unimportant/important”; “non-beneficial/beneficial”; “not fond of/fond of”; “not enjoyable/enjoyable”; “bad/good”; “unpleasant/pleasant”, and “unfavorable/favorable”. The reliability of the combined scale was very high (α = 0.96). Attitude was then computed into a variable which contained the mean score of all ten items (M = 3.93, SD = 1.17).

#### 2.4.2. Interest in Healthy Food 

The moderating variable *interest in healthy food* was measured through modifying a scale adopted by Pratt [40], which was originally designed to measure involvement with wine (from 1 = *strongly disagree* through 7 = *strongly agree*). The seven items were adapted to the following: “I am very interested in healthy foods”, “I find conversations about healthy foods very enjoyable”, “I wish to learn more about healthy foods”, “Deciding which healthy foods to buy is an important decision”, “I consider healthy foods to be a central part of my lifestyle”, “For me, eating healthy foods is a pleasurable experience”, and “Healthy foods are enjoyable to consume socially”. The scale used for measuring consumers’ interest in healthy food had a high internal consistency (α = 0.92) and can therefore be considered reliable [41]. Interest in healthy food was then computed into a variable which contained the mean score of all seven items (M = 4.84, SD = 1.14). 

#### 2.4.3. Buying Intention 

The dependent variable *buying intention* was measured with the combination of two scales. The first scale, developed by Yi [42], consisted of three bi-polar items that were based on the question: “How likely is it that you would consider buying this product the next time?” The participants could answer on a 7-point-scale with “unlikely/likely”; “impossible/possible”, and “improbable/probable”. The second scale, adapted from Baker & Churchill [43] consisted of three statements about how likely the participants would consider purchasing the product upon their next visit: “I would like to try this product”; “I would buy this product when I would see it in the store”, and “I would actively seek out this product in the store in order to purchase it”. The participants could agree with these statements on a 7-point Likert scale (1 = strongly disagree, 7 = strongly agree). The reliability of the total buying intention scale was good (α = 0.93). Buying intention was then computed into one variable which contained the mean score of all six items (*M* = 3.39, *SD* = 1.47). There were no indications of respondent fatigue, and the factor analysis and reliability analysis did not provide any indication of problems. We further checked for straight liners and we asked at the end of the questionnaire whether participants wanted to comment, but no comments were related to this point.

### 2.5. Statistical Analysis 

Before testing our hypotheses, we conducted randomization checks with a 1-factor ANOVA for gender, age, and interest in healthy food. Gender was not included in the ANOVA. We have also conducted an ANCOVA with gender as covariate, but no different effects were found. Table 1 presents the means and standard deviations for all variables separately for each condition. No differences were found between the conditions, suggesting randomization was successfully conducted. We computed residual scores and tested them for Mahal’s distance, Cook’s distance, and leverage scores, and found no indication that would confirm the assumption of outlying scores. Subsequently, Pearson’s correlations between the model variables were calculated, but none of the variables correlated with attitude towards the product (*p* > 0.05) or buying intention of the product (*p* > 0.05), so none of these factors were used as covariates in the causal analyses. Attitude towards the product correlated significantly with buying intention of the product (*r* = 0.741, *p* = 0.000), and gender correlated significantly with interest in healthy food (*r* = 0.220, *p* = 0.000). 

Univariate analysis of covariance (ANOVA) tested the effect of the experimental conditions on attitude towards the product. Subsequently, an ordinary least regression analysis was conducted to examine the relation between attitude towards the product and buying intention. The adjusted *p* value that was considered significant was 0.05. We calculated effect sizes for Cohen’s f, with values of 0.02, 0.15, and 0.35 indicating small, medium, and large effect sizes, respectively. To further test for the (non-) existence of the main effects of the experimental condition [44], Bayesian ANOVA was performed with the statistical program JASP. Evidence for each model in this analysis was evaluated against the null model. Following conventional interpretation, a value of BF10 above 3 is interpreted as substantial support for the alternative hypothesis, and a value of BF10 less than 0.33 as substantial support for the null hypothesis. BF10values between 0.33 and 3 suggest the data are insensitive [43]. A linear regression with covariates has also been conducted, but no significant effects were found in these analyses as well (*p* > 0.05). Therefore, we remained with the original ANOVA. 

## 3. Results

Prior to testing the hypotheses, descriptive statistics were used to report frequencies of gender, age, interest in healthy food, attitude towards the product, and buying intention, used in the present research, split out per condition as demonstrated in Table 1.

### 3.1. The Effect of Health Claims on Food Packaging on Attitude

The results of an ANOVA showed that attitude towards the product did not significantly differ between the color conditions, F(1,296) = 0.567, *p* > 0.05) and health claim conditions, F(1,296) = 0.155, *p* > 0.05. Bayesian ANOVA was consistent with these results and supported evidence against the effect of the color (BF10 = 0.167) and claim (BF10 = 0.137), and for the interaction (BF10 = 0.039). Therefore, we reject hypothesis H1a and H1b. Moderation analysis showed no moderating effect of interest in healthy food and color (*p* > 0.05) and claim (*p* > 0.05). 

### 3.2. The Effect of Attitude Towards the Product on Buying Intention 

A simple linear regression was calculated to predict buying intention based on attitude towards the product. Attitude towards the product significantly predicted intention to buy, b = 0.741, t(298) = 19.075, *p* < 0.001. Attitude towards the product also explained a significant proportion of variance in buying intention, R2 = 0.741, F(1,298) = 363.8, *p* < 0.001. These results confirmed H3.

## 4. Discussion

Inspired by trends towards health issues, concerns for negative consequences of unhealthy food consumption [5] and proliferation of BFY foods that contain high levels of sugar, use harmful substitutes for sugar or have a very low nutrition value [6,10], this study aimed to investigate the effect that health claims on food packaging have on the attitude towards the product and on the buying intention of this product. 

Based on the literature that describes the influence of packaging features on product evaluations e.g., [14,26,28], it was expected that participants confronted with a product with a low-arousal packaging color would have a more positive attitude towards the product, compared to participants that encountered a product with high-arousal packaging colors. It was also expected that participants that saw a product with a palatability claim would have a more positive attitude towards the product, in comparison to participants that encountered a product with a health claim. Both predictions could not be confirmed by the experiment. The results thus do not support the assumption that visual cues (color) and textual cues (health-related advertising claims) are effective in conveying symbolic meaning about the product’s healthfulness and consequently, influence consumer attitude towards the product [13]. 

If we look at the influence of health claims on food packaging altogether (e.g., the combined effect for visual and textual cues), results showed no significant effect from health claims on buying intention as well. A possible explanation for these results is that participants did not recognize the symbolic meaning of the products’ healthiness or tastiness that was communicated through the deployment of different packaging colors and advertising claims. The absence of an effect might also be grounded in the use of the scenario. Participants were asked to imagine themselves shopping in the dairy isle at the local supermarket where they encountered the product (stimulus) presented. They were not actively involved in the scenario or tested the product they were exposed to, so in real-life results might be different, although there is an increasing number of people conducting their grocery shopping online. 

In addition, the effect of the attitude towards the product on buying intention was examined. It was expected that participants with a more positive attitude towards the product would have a stronger buying intention than participants with a less positive attitude towards the product. The results from the current study confirmed this assumption, showing a significant effect from attitude on buying intention, in accordance with assumptions grounded in the theory of planned behavior [25]. 

It would be advisable for other researchers to keep in mind that packaging design incorporates many different elements, and that many of these elements can operate as cues in consumer evaluations of a products’ healthfulness. Also, future studies should test the effect of health claims on buying intention with more types of BFY products or food groups, i.e., not limit participants in their freedom of choice, and to see how their perception of BFY foods differs from “palatable” foods. The reaction towards different stimuli presented via different types of palatable foods may differ. 

One of the strengths of the current study is the large number of participants that were included. Bayesian analyses has shown that the number of participants has been enough to analyze the hypotheses that were stated. Secondly, most studies in food marketing have studied the effects of unhealthy food marketing, especially targeting children [2,45]. However, in order to be better able to improve dietary intake among people, more research is needed to examine how people can be motivated to eat healthier. The present study adds to the literature in the field, by examining how food packaging can influence healthier food consumption patterns, although the results were not significant. Thirdly, although most researchers acknowledge that media and communication messages do not affect everyone in the same way, moderation analyses are not often conducted [2,46]. The current study examined whether interest in food was moderating the effect of healthy and unhealthy food packaging. 

In contrast, the study also has a number of limitations concerning reliability and generalization of results. First, whereas different studies have shown that intention is a strong predictor for actual behavior [25,47,48,49,50], a limitation of this study was that actual buying behavior could not be measured. Even though using buying intention as a dependent measure could provide us with useful insights about how participants react to a stimulus, it might be less strong than measuring actual in-store behavior, because intention may not always result in behavior. For instance, responses might be different on another point in time, because of the occurrence of unforeseeable events or changes in dietary intake. It might therefore be interesting to repeat this experiment in a more realistic shopping environment where actual behavior can be measured. Secondly, in the present study, we tested the effect of only two types of packaging cues, namely color (visual cues) and claims (textual cues), on attitude and buying intention. 

Previous research suggests that both packaging cues tested are able to convey symbolic meaning about the product’s healthiness. Color and nutrition content claims can influence consumer’s perceptions of food healthiness and purchase intention [51,52]. More specifically, it is suggested that marketers try to capitalize the association between green and health [53]. For example, it has been shown that color influences perceptions of a product’s healthfulness [52], whereby results demonstrated that green labels increase perceived healthfulness, especially among consumers who place high importance on healthy eating. A different study [53] on the other hand found only little effect of health and nutrition claims on consumer judgments of food healthfulness. However, other packaging design factors, such as layout, shape, logo design, illustrations, use of white space, and typeface might also contribute to product perceptions related to healthiness [14,27,28,29,34]. Future research could further pinpoint the role of health cues on packaging design in relation to consumers’ healthiness perception. In addition, consumer’s knowledge about high sugar levels in fruit yoghurt, or perceptions of the food packaging (e.g., if there was sugar-added, no sugar, or artificially sweetened products) may have affected their attitude towards the product and interacted with the attitude responses. These factors were not assessed in the current study. Because we used large groups of participants and randomized the participants over the conditions, it can be expected that these potential confounding variables are evenly distributed over the conditions and do not affect the results. Nonetheless, future studies could control for these factors. 

Thirdly, the study relied solely on data from an online questionnaire. Internet usage for survey research has provided several advantages compared to more traditional methods of data collection [54]. One of the most reported advantages is efficacy related to time and money. Online data collection generally takes comparatively less timely and is a less expensive means for measuring attitudes, opinions and behaviors [54]. In addition, participants in online surveys may be less prone to providing more socially desirable information and observable behaviors caused by the presence of the researcher. Individuals have a better sense of anonymity, leading to a decreased likelihood of response bias and increased response rate [55]. Although online questionnaires are a cost-effective, accessible, and quick way to gather insights about consumer attitudes and preferences, it should be noted that participants may have given socially desirable answers. For instance, people may have answered questions about “interest in healthy food” in a socially desirable way due to the overall trend towards a healthier lifestyle. Similarly, they may have categorized themselves according to subjective rather than objective self-perception. This influences the validity of research data [56]. We have tried to ensure unbiased responses by guaranteeing a high level of anonymity and by putting emphasis on the fact that there are no right-or-wrong answers. 

Future research could overcome some of these limitations by expanding the current research with a variety of neurophysiological approaches (physiological and psychological measures), such as pupillary response, heart rate, eye movements, voice pitch analysis, and neuroimaging [57]. For instance, eye tracking could be used to measure visual attention to cues on food packaging [58]. Other possible physiological measures could include heart rate, galvanic sweat response, and respiration rate. These measures should decrease the chances for socially desirable answering and provide us with more knowledge about people’s unconscious responses to packaging and labels. Future research should further focus on potential moderating factors, e.g., attitude toward palatability claims, which could influence the effect. 

## 5. Conclusions

The study could not confirm if health cues on food packaging, such as color and advertising claims, had an effect on consumers’ attitude towards the product and hence, their intention to purchase the product. In contrast to health cues on food packaging, consumers’ attitude towards the product did, however, have a significant effect on buying intention. We have seen that consumers do not react differently to health cues than they do to cues related to palatability, and that none of the two packaging cues affect their intention to purchase the product. Instead, buying intention is explained mostly by whether consumers have a positive or negative attitude towards the product: when participants had a more positive attitude towards the product, they were more likely to say that they intended to purchase the product. These findings confirm other theories stating that people’s attitudes are powerful predictors of buying decisions. 

## Figures and Tables

**Table 1:** Variables measured by condition (mean and standard deviation).

	Low-Arousal Colors, Nutrition/Health Claim (*n =* 73)	Low-Arousal Colors, Palatability Claim (*n =* 74)	High-Arousal Colors, Nutrition/Health Claim (*n* = 78)	High-Arousal Colors, Palatability Claim (*n =* 75)	Differences	Total
Gender (male)	49 %	54 %	41%	56%	*p* > 0.05	50 %
Age (y)	34.9 ± 13.4	36.4 ± 12.3	36.5 ± 13.6	37.4 ± 12.9	*p* > 0.05	36.4 ± 13.01
Interest healthy food	4.5 ± 1.2	4.9 ± 0.9	5.0 ± 1.1	4.9 ± 1.2	*p* > 0.05	4.8 ± 1.1
Attitude product	3.9 ± 1.1	4.0 ± 1.2	3.9 ± 1.2	3.9 ± 1.2	*p* > 0.05	3.9 ± 1.2
Buying intention product	3.4 ± 1.4	3.6 ± 1.5	3.3 ± 1.5	3.3 ± 1.4	*p* > 0.05	3.4 ± 1.5
